# Supplementary material for: A 3D hybrid-shot spiral sequence for hyperpolarized 13C imaging
Source: Magn Reson Med. Author manuscript; Available in PMC 2021 Jul 23. (PMC7611357; doi:10.1002/mrm.28462)
Supplement: Appendix A [file EMS130857-supplement-Appendix_A.pdf]

APPENDIX A
HSS READOUT ALGORITHM

Pseudo-code, describing the algorithm used to generate the spiral trajectories in this work, is shown below. The algorithm was modified from an existing VDS design algorithm[29] and implemented in the C programming language

$N \leftarrow N;$

$\ddot{k}_r, \ddot{\theta}, \dot{k}_r, \dot{\theta}, k_r, \theta \leftarrow 0;$

while $k_r < k_{trans}$ **do**

  calculate next $\ddot{k}_r, \ddot{\theta}, \dot{k}_r, \dot{\theta}, k_r, \theta;$

end

$\dot{k}_{\text{trans}} \leftarrow \dot{k}_r;$

$\ddot{k}_r, \ddot{\theta}, \dot{k}_r, \dot{\theta}, k_r, \theta \leftarrow 0;$

$N \leftarrow 1;$

while $k_r < k_{trans}$ **do**

  calculate next $\ddot{k}_r, \ddot{\theta}, \dot{k}_r, \dot{\theta}, k_r, \theta;$

  output $\ddot{k}_r, \ddot{\theta}, \dot{k}_r, \dot{\theta}, k_r, \theta;$

end

$N \leftarrow N;$

$\dot{k}_r \leftarrow \dot{k}_{\text{trans}};$

while $k_r < k_{max}$ **do**

  calculate next $\ddot{k}_r, \ddot{\theta}, \dot{k}_r, \dot{\theta}, k_r, \theta;$

  output $\ddot{k}_r, \ddot{\theta}, \dot{k}_r, \dot{\theta}, k_r, \theta;$

end

The algorithm begins by calculating $\dot{k}_{\text{trans}}$ (the first while loop), before resetting and calculating the single-shot trajectory up to $k_r = k_{\text{trans}}$ (the second while loop), at which point N is set to the number of shots in the multi-shot region and $\dot{k}_r$ is set to $\dot{k}_{\text{trans}}$. After which, the algorithm proceeds to calculate the multi-shot portion of the trajectory (the third while loop).
